# Maternal treadmill exercise ameliorates impairment of neurological outcome, caspase-1 and NLRP3 gene expression alteration in neonatal hypoxia-ischemia rats

**DOI:** 10.22038/IJBMS.2022.66183.14544

**Published:** 2023-02

**Authors:** Elahe Gorgij, Hamed Fanaei, Parichehr Yaghmaei, Mohammad Reza Shahraki, Hadi Mirahmadi

**Affiliations:** 1 Department of Biology, Science and Research Branch, Islamic Azad University, Tehran, Iran; 2 Pregnancy Health Research Center, Zahedan University of Medical Sciences, Zahedan, Iran; 3 Department of Physiology, Zahedan University of Medical Sciences, Zahedan, Iran; 4 Infectious Diseases and Tropical Medicine Research Center, Zahedan University of Medical Sciences, Zahedan, Iran

**Keywords:** Brain hypoxia-ischemia, Caspase-1, Infant, NLRP3, Pregnancy, Rats, Treadmill

## Abstract

**Objective(s):**

Neonatal hypoxia-ischemia (HI) is one of the most important causes of neurological disorders in children. Various studies suggest that maternal exercise during pregnancy has a beneficial impact on the health status of offspring infants. In this study, the effect of maternal treadmill exercise during pregnancy on neurological and molecular changes induced by HI in newborn rats was investigated.

**Materials and Methods:**

In this experiment, 24 pregnant female rats were divided into two groups; the first group was subjected to treadmill exercise for six weeks. The treadmill exercise program was initiated by running for 17 min at 5–10 m/min at 0 inclination in the first week, followed by running for 21 min at 5–25 m/min at 5° inclination in the second week, running for 25 min at 5–30 m/min at 10° inclination in the third and fourth weeks, running for 25 min at 5–15 m/min at 10° inclination in the fifth and sixth weeks. The second group was left untreated and did not perform the exercise. Newborn rats were assigned to four groups; (1) control, (2) control+exercise, (3) HI, and (4) HI+exercise. HI was developed in the offspring on the 8th postnatal day. One week following the induction of HI, the Garcia test was carried out. The histological morphology of neonates was assessed, and the expression levels of caspase-1 and NLRP3 were evaluated.

**Results:**

The data showed that maternal exercise during pregnancy significantly improved neural cell death (*P*<0.001) and the Garcia score (*P*<0.05), while it attenuated the expression levels of caspase-1 (*P*<0.001) and NLRP3 (*P*<0.05) genes in newborn rats induced by HI.

**Conclusion:**

These results demonstrated that maternal treadmill exercise during pregnancy could reverse the neurological deficits, as well as the expression levels of caspase-1 and NLRP3 genes, which occur in neonatal hypoxia-ischemia.

## Introduction

Neonatal hypoxia-ischemia (HI) leads to encephalopathy that impairs movement and memory (1, 2). Behavioral changes can be directly related to volume reduction and atrophy of specific brain structures (3, 4), associated with cytotoxicity, oxidative stress, and apoptosis (5). HI impairs brain function and causes a series of neurological defects, mainly affecting motor and sensory functions as well as body symmetry and coordination (6).

The family of Caspase proteases is divided into two main subfamilies, one of which is involved in inflammation (caspase-1, 4, 5, 11, 12, and 13) and the other in apoptosis (caspase-2, 3, 6, 7, 8, 9, 10, and 14) (7, 8). Caspase-1 mediates neuronal cell death during HI. Caspase-1 is activated by the nucleotide-binding oligomerization domain, leucine-rich repeat, and pyrin domain-containing (NLRP) cascade and causes programmed cell death (9, 10). Previous studies have provided evidence for the role of caspase-1 in cell death and cerebral ischemia *in vitro* and *in vivo* (11, 12). 

Inflammasome NLRPs have recently been implicated in the inflammatory response during ischemia (13). NLRP3 is an essential receptor of the innate immune system, and its activation induces many pro-inflammatory cytokines that play a role in the development of various diseases (14). NLRP3 is a cytosolic macromolecular complex consisting of the Nlrp1/3 receptor, caspase-1, and an adaptive protein that facilitates interaction between them (15). The proximity-induced auto-activation of NLRP3 converts the precursor caspase-1 into the cleaved caspase-1 (16). The cleaved Caspase1 causes inflammatory reactions and subsequently leads to cell death (17). 

Cerebral hypoxia-ischemia in neonatal rats was induced by occlusion of the right carotid artery, followed by hypoxic conditions resulting in generalized alterations in motor-sensory function. Rice *et al*. (14) presented a modified Levin model (18), utilizing 7-day-old rats subjected to right carotid occlusion with a surgical suture, followed by a period of hypoxia (8% oxygen and 92% nitrogen) to analyze the processes involved in hypoxic-ischemic brain injury. This model was used by researchers to assess the impact of perinatal hypoxia on molecular, morphological, behavioral, and biochemical changes occurring during ischemia (5).

Effective therapy to prevent or treat the consequence of HI is still far from available. Aerobic exercise is widely acknowledged as an effective treatment for managing chronic conditions such as obesity, heart disease, type 2 diabetes, and osteoporosis (19, 20). Exercise can lead to neurogenesis, cell proliferation, increased synaptic plasticity, and modulated inflammatory responses (21, 22). Treadmill exercise was reported to improve hypoxia-ischemia-induced sensory-motor impairment in neonatal rats (23, 24). It has been reported that maternal-voluntary wheel running exercise during pregnancy prevents the loss of HI-induced hippocampal neurons in offspring (25, 26). Maternal swimming significantly increased learning and memory in the Morris Water Maze test, as well as the number of neurons in the hippocampus of rat neonates (27, 28). Moreover, we recently showed that treadmill exercise during pregnancy of obese women improved infarct volume, edema, and neurobehavioral function, as well as modulating the expression levels of Bax, Bcl-2, and IL-6 genes, in a rat model of HI (29, 30).

As previously stated, treadmill activity during pregnancy may improve neurological function in HI-induced rat neonates; however, there is no evidence of pathogenic or inflammatory alterations in these neonates. Given the importance of caspase-1 and NLRP3 in inflammatory responses, we used the Garcia score to measure neurological function and examined caspase-1 and NLRP3 gene expression levels in this investigation. Pathological changes caused by HI were also examined in the brains of newborn rats.

## Materials and Methods

***Experimental animals and exercise protocols***

24 female Wistar rats (6 to 8 weeks old, weight 200 ± 20 g) from the Laboratory Animal Research Center of Zahedan University of Medical Sciences were used. Rats were housed in cages under controlled light/dark (12/12 hr) and temperature (23 ± 3 °C) conditions and were provided with food and water *ad libitum*. They were adapted to laboratory conditions for 1 week before experiments began. This study was approved by the animal ethics committee of the institute (Ethical code: IR.ZAUMS.REC.1399.514). These animals were then placed next to the male animals (10 heads) (two females and one male in each cage). By observing the vaginal plaque, the zero-day pregnancy was determined. Then, the animals were divided into two groups: 1 - They did not exercise (from the neonates of this group control and hypoxic ischemia groups were selected) 2 - They exercised daily during pregnancy (pups of these animals were used for the hypoxia-ischemia + exercise group and the control + exercise group). These animals were regularly trained according to the running protocol (Table 1) for 6 weeks (29-32).

After birth, the pups were divided into 4 groups:

1. Control group: In this group, surgery was performed, but the blood flow to the brain was not stopped, and also hypoxia was not induced.

2. Hypoxia-ischemia group: Animals in this group underwent surgery, and blood flow to the right carotid artery was blocked. They were also exposed to 8% oxygen for 90 min.

3. Control + Exercise group: In this group, surgery was performed, but the blood flow to the brain was not stopped, and also hypoxia was not induced.

4. Hypoxia-ischemia group + exercise: Animals in this group underwent surgery, and blood flow to the right carotid artery was blocked. Also, they were exposed to 8% oxygen for 90 min.

***Cerebral hypoxia-ischemia induction***

To prepare the animal for cerebral ischemia, 8-day-old pup rats were first anesthetized with 2.5% isoflurane (Isoflurane, UK). The body temperature was maintained at 36–38 °C, while the surgical procedure was performed. After fixing the animal on the operating table, a midline incision was performed, and the right common carotid artery (CCA) and its branches (external and internal) were exposed when the connective tissue and muscles were retracted. Then, by temporarily closing the CCA on the right side, created a fine gap in the external carotid branch and at the same time, a monofilament nylon thread with a heat-rounded tip was used to occlude the middle cerebral artery. It was inserted through the incision into the internal artery and from there slowly into the skull and toward the Willis ring to reach the beginning of the middle artery of the brain. One hour after the offspring recovered, they were exposed to 8% oxygen for 1.5 hr. CO_2_ adsorbent was also used to remove the acid-causing factor inside the chamber.

***Garcia behavioral assessments***

To evaluate neurological outcomes after induced ischemia, the Garcia behavioral assessments were performed 7 days after the stroke. According to the study of Garcia *et al*. (1995), the following six behaviors were evaluated: (1) spontaneous activity that was monitored for 5 min, (2) assessment of the symmetry of the four limbs during the movement of animals, (3) evaluation of the symmetry of forelimb outstretching, (4) capability of climbing, (5) proprioception of the body, and (6) refection to vibrissae touch. Each behavior was ranked based on a scale between 0 and 3 points: Normal 3, slightly affected 2, severely affected 1, and no movement 0.

***RT- PCR assay***

The rnx-plus kit was utilized to extract total hippocampal RNA (SinaClon, Iran), according to the manufacturer’s instruction, and afterward, the isolated RNA was dissolved in DEPC-treated water. All isolated samples were quantitated by a NanoDrop-1000 UV–vis spectrophotometer (Midland, ON, Canada), and a value of ~2.0 for optical density (OD) 260/280 nm ratios was considered pure for RNA samples. Furthermore, the electrophoresis of isolated RNA was performed in a 1.5 % gel of agarose. RT-PCR was utilized to evaluate Caspase- 1 and nlrp 3 expression levels using the Pars Genome’s RNA amplification Kit (Pars Genome, Iran). 

Briefly, the single-strand cDNA synthesizes of Caspase- 1, NLRP 3, and GAPDH (reference gene) were carried out using the oligo-dT primer and M-MuLV enzyme. The primers of RT- PCR were as follows: 

The extracted RNAs were elongated by poly (A) polymerase enzyme (PAP) in the first step. The reaction was achieved in a total volume of 20 μl including 1.5 μg of RNA with 0.4 μl of PAP, 2 μl of 10x buffer, and 1 μl of ATP at the temperature of 37 °C for 20–30 min. After that, 5 μl of the above samples were mixed with 5x buffer, dNTP, 0.3− 0.4 μl M-MuLV enzyme, and 0.3 μl of RNase inhibitor (Ribolock). The tubes were placed at 42 °C for 60 min and 80 °C for 3 min (to inactivate the RT enzyme). 2X SYBR Green master mix of PCR (Parstoos, Iran), cDNA products, specific primers (Pars Genome, Iran), and DEPC-treated water were used to perform RT-PCR. The data analysis was performed by comparing Caspase- 1 and NLRP3 with the GAPDH expression level for each sample.

***Cresyl violet staining***

*Tissue and molding process*

In general, in order to keep the cells and tissues of the body in a similar state and close to their living state, a stabilization operation is performed. After removing the sample from the living organism, it is immediately placed in a 10% formalin solution for 24-72 hr. Due to the high percentage of water in the tissue, paraffin does not penetrate the tissues, so dehydration with ethyl alcohol should be done. Dehydration with ethanol starts as follows from low degrees to reaching absolute alcohol: Alcohol 70 (50% min), Alcohol 80% (50% min), Alcohol 90 (50% min), Alcohol 100 (50% min). The presence of alcohol in paraffin causes problems, so it is better to remove the alcohol from the environment with a mixture that reacts with alcohol on one side and paraffin on the other, and they are related to each other. For this purpose, a solution called xylol was used. After xylol, paraffin is used to harden and prepare the tissue for molding and cutting tissue: Xylol 1 (50 min), Xylol 2 (50 min), Paraffin 1 (50 min), Paraffin 2 (50 min). Samples were cut by a 5 μm thick microtome machine and placed on a fluidized sieve.

*Cresyl violet (Nissl) procedure*

Nissl substance is found in fixed neurons and includes the granular endoplasmic reticulum and ribosomes occurring in the soma and dendrites. For staining at first, the slides are deparaffined and hydrated. The samples are then placed in 0.1% cresyl violet acetate solution (0.1 g cresyl violet (Sigma-C5042) and 0.3 ml acetic acid glacial (Merck-1.00056)) for 10 min at a temperature of 37-50 °C. After that, rinsing with distilled water and soaking in 95% alcohol for 2-3 min is performed. The slides were then washed in water and after drying, the lamel was pasted on the sample and photographed with a light microscope (LABOMED).

***Statistical analysis***

 All the statistical analyses were done using IBM SPSS Statistics 22 software (Chicago, IL, USA). The data distributions for normality and equal variances were checked using the Shapiro–Wilk and Levene’s tests, respectively. Data were expressed as mean ± standard error of the mean (SEM). The means for different groups were compared using one-way ANOVA. Statistically significant differences were further assessed by Tukey *post hoc* tests. *P*<0.05 was considered significant.

## Results

***Effects of ischemia-hypoxia and exercise on pathological alterations***

**Figure 1:**
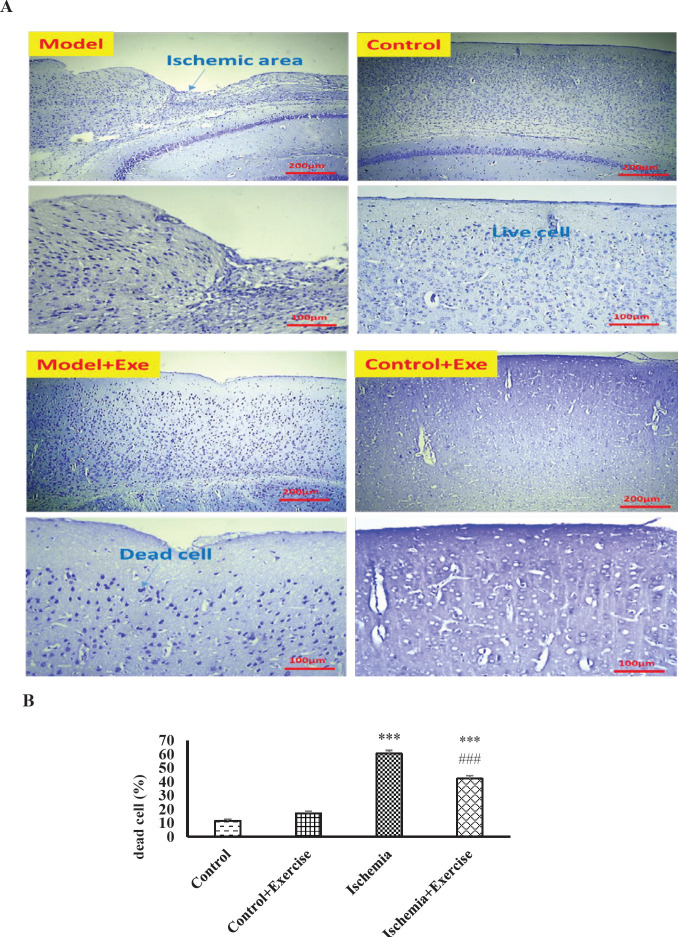
Effect of hypoxia-ischemia and maternal treadmill exercise on dead cells and live cells was represented by cresyl violet staining (A) and the percentage of dead cell chart (B). Data are shown as mean±SEM (n = 4). ***P<0.001 versus the control group. ###*P*<0.001 as compared with the hypoxia-ischemia group

Cells whose DNA has been destroyed, absorb more cresyl or Nissl dye and turn dark blue or dark purple. With IMAGE J software, the number of dead cells was counted. The cresyl violet staining (Figure 1A and B) revealed that ischemia-hypoxia significantly increased the brain-dead cells as compared with the control group (*P*<0.001). Exercise significantly improved cell death induced by ischemia compared with the ischemia-hypoxia group (*P*<0.001).

***Effects of ischemia-hypoxia and exercise on Garcia’s score***

**Figure 2:**
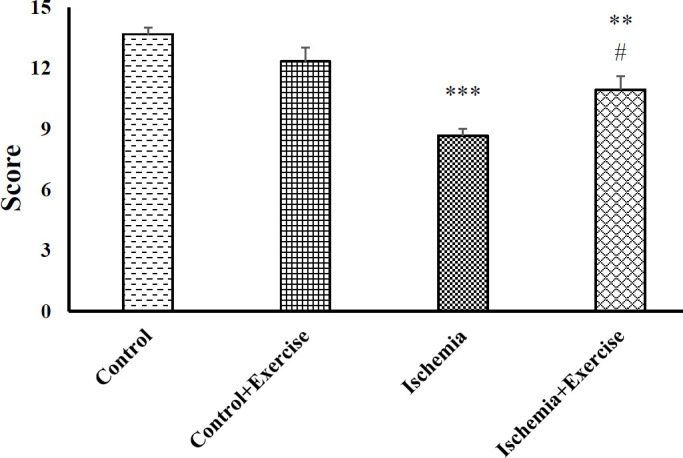
Effect of hypoxia-ischemia and maternal treadmill exercise on the Garcia score. Data are shown as mean±SEM (n=7). ***P<0.001 and **P<0.01 versus control group. #*P*<0.05 as compared with the hypoxia-ischemia group

The Garcia behavioral test was performed to determine the neurological deficit in the offspring rats receiving ischemia–hypoxia injury by CCA occlusion and the effect of exercise on it. There were not any significant differences between the control and control+ exercise groups (*P*˃0.05). The ischemia–hypoxia group demonstrated significantly lower Garcia scores than the control group (*P*<0.001, Figure 2). Exercise improved the neurological deficit scores in the ischemia–hypoxia + exercise group (*P*<0.05). 

***Effects of ischemia-hypoxia and exercise on caspase-1 mRNA level***

**Figure 3:**
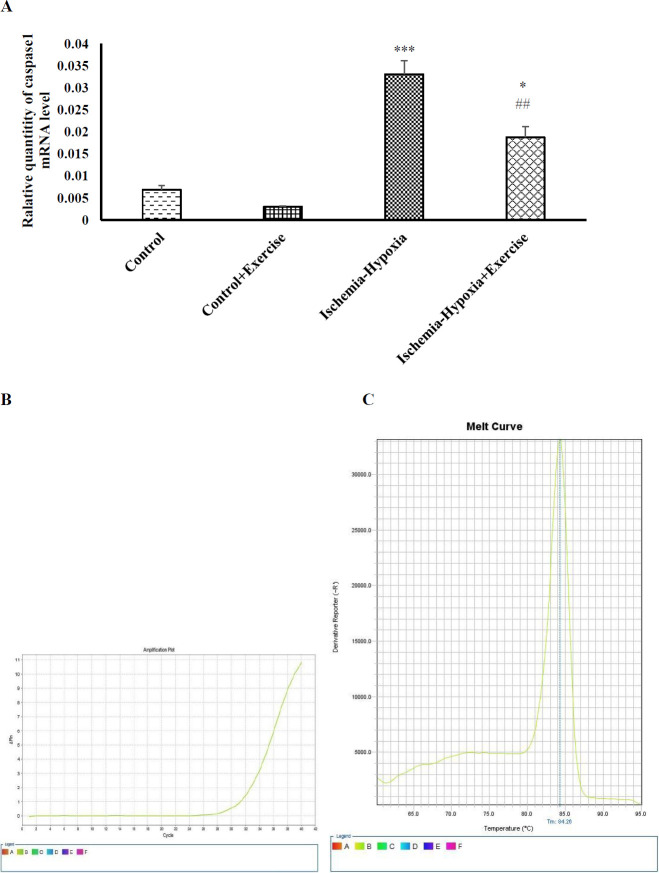
Effect of hypoxia-ischemia and maternal treadmill exercise on (A) caspase1 mRNA expression level. The amplification (B) and melting (C) curves of one case are represented. The mRNA expression level was measured by RT-PCR and normalized to GAPDH levels. Data are shown as mean±SEM (n=7). **P<0.01 versus the control group. #*P*<0.05 as compared with the hypoxia-ischemia group

As shown in Figure 3A, ischemia–hypoxia significantly increased the mRNA level of caspase-1 in ischemia–hypoxia and ischemia–hypoxia + exercise groups as compared with the control group (*P*<0.001 and *P*<0.05, respectively). Exercise significantly decreased the mRNA level of caspase-1 in the ischemia–hypoxia + exercise group compared with the ischemia–hypoxia group (*P*<0.01, Figure 3A). The amplification plot (B) and melting curve (C) of one sample for caspase-1 are reported. The melting curve analysis was done to confirm the specific amplification.

***Effects of ischemia-hypoxia and exercise on NlRP3 mRNA level***

**Figure 4:**
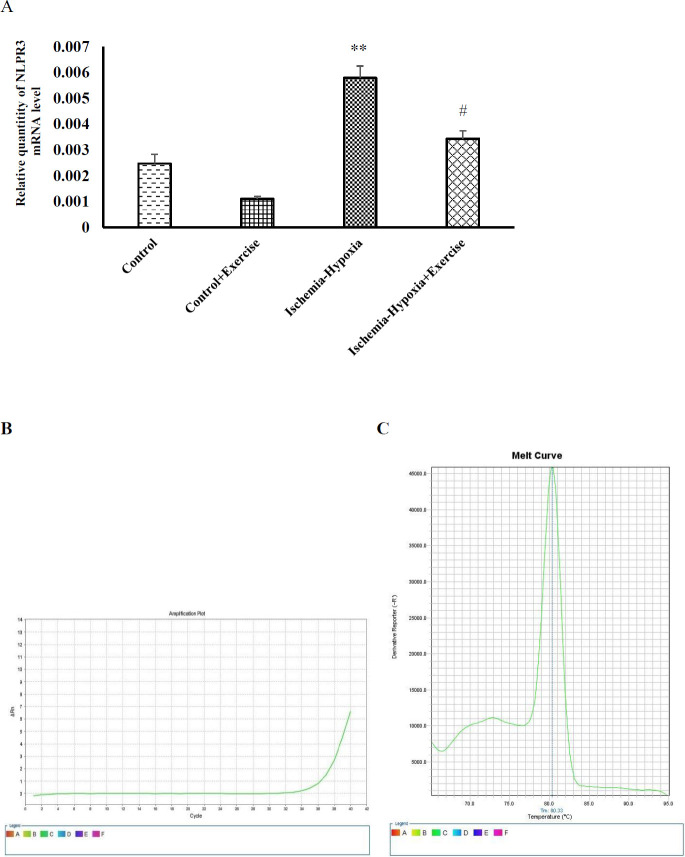
Effect of hypoxia-ischemia and maternal treadmill exercise on (A) nlrp3 mRNA expression level. Amplification plot (B) and melting curve (C) of nlrp3 are represented. The mRNA expression level was measured by RT-PCR and normalized to GAPDH levels. Data are shown as mean±SEM (n=7). ***P<0.001 and *P<0.05 versus the control group. ##*P*<0.01 as compared with the hypoxia-ischemia group

The neural Nlrp3 levels were significantly increased in the ischemia–hypoxia rats as compared with the control group (*P*<0.01, Figure 4A). Six-week treadmill running significantly decreased the NLRP3 levels in the ischemia–hypoxia + exercise as compared with the ischemia–hypoxia group (*P*<0.05). The amplification plot (B) and melting curve (C) of one sample for NLRP3 are shown. To confirm the specificity of the amplification, a melting curve analysis was done.

## Discussion

We previously demonstrated that maternal exercise during pregnancy reduces infarct volume, boosts Bcl-2 and BDNF expression, and lowers Bax and IL-6 expression (29, 30). The role of maternal exercise in the modulation of neonatal ischemia-hypoxia was investigated, and tissue changes, as well as inflammatory changes, were analyzed in this study. According to present data, maternal exercise improved neural cell death and the neurological Garcia score while it mitigated the expression levels of caspase-1 and NLRP3 genes in hypoxia-ischemia-induced rats.

Physical activity has many metabolic benefits. It has been documented that maternal exercise can reprogram the fetal metabolic profile (27), potentially altering an individual’s susceptibility to diseases in adulthood (33). In this study, we developed a hypoxia-ischemia model to evaluate the effect of maternal treadmill exercise during pregnancy on neonatal cell death, neurological disorders, and altered caspase1 and NLRP3 gene expression levels. The results of the crystal violet test indicated reduced neural cell death caused by hypoxia-ischemia in newborn rats after maternal treadmill exercise during pregnancy.

It has been reported that the Garcia score can predict infarct size ≥30% (34). The Garcia test shows the broadest spectrum of neurologic deficits and probably reflects anatomical neurologic function. Moreover, a strong correlation was observed between ischemia and the Garcia test (35). It has been reported that aerobic exercise improves behavioral changes and neurocognitive disorders and induces hippocampal neurogenesis (36-38). In addition, it has been indicated that treadmill exercise led to decreased DNA fragmentation in animal models induced by HI (23, 28, 39). Besides, Akhavan *et al*. (40, 41) showed that hippocampal neuron loss in animal pups induced by HI was prevented by maternal voluntary wheel running exercise during pregnancy. Improvements in Morris water maze performance and neuron numbers in the hippocampus of neonatal HI rats have been shown after maternal swimming. (25, 27). In line with these documents, the findings of the present study revealed that HI-induced rat pups showed decreased spontaneous activity, symmetry in the movement of the limbs-tail suspension, forepaw extension, climbing the wall of a wire cage, body proprioception, and response to vibrissae touch according to the Garcia test. Accordingly, maternal treadmill exercise significantly improved these items in the Garcia score in HI-induced newborn rats.

Exercise is a valuable intervention method for modulating innate immune responses (42). Previous research has shown that chronic endurance training, resistance training, and high-intensity interval training all reduce NLRP3 inflammasome activation in response to numerous clinical conditions. Vandanmagsar *et al*. (43) reported that exercise significantly reduced the mRNA expression levels of IL-1β and NLRP3 genes in the subcutaneous tissue of patients with type 2 diabetes. Studies suggested that moderate-intensity treadmill training for four weeks significantly reduced the activation of NLRP3 inflammation in mice and rats in adipose tissue (44, 45), liver (46, 47), hippocampus (48-50), prefrontal cortex (51), and substantia nigra (52) induced by various diseases. These results are consistent with our findings, which found that maternal chronic mild-intensity treadmill exercise reduced caspase-1 and NLRP3 gene expression levels in HI-induced animal models. However, some studies reported that chronic exercise with high intensity as well as acute exercise increased NLRP3, IL-1β, and IL-18 gene expression levels (42, 53, 54). This discrepancy in the expression of the NLRP3 gene may depend on the type of training, the chronic or acute exercise performance, and also the intensity of the exercise performed. Thus, chronic moderate-intensity training decreases the expression, while acute exercise or chronic high-intensity exercise can increase the expression of these inflammatory factors.

It has been demonstrated that HI enhanced Caspase-3 expression in the dentate gyrus, while treadmill exercise significantly decreased its expression (55). In this study, a hypoxia-induced increase in neural caspase-1 gene expression levels in neonates was partially restored by maternal treadmill exercise during pregnancy. According to previous research, maternal treadmill exercise during pregnancy increases anti-oxidant capacity and BDNF levels (30), while decreasing pro-inflammatory cytokine production, NF-κB expression (56), and IκB-α phosphorylation. Maternal exercise suppressed Bax expression (57) and the level of Caspase-3, whereas it enhanced the expression of Bcl-2 and pAkt in newborn rats. Consequently, maternal treadmill exercise-induced caspase-1 and NLRP3 down-regulation may also contribute to the protective effects of maternal treadmill exercise during pregnancy against neonatal hypoxia-ischemia.

**Table 1:** Six weeks maternal treadmill exercise protocol during pregnancy in rat

**r-Nlrp3-f**	GGAGTGGATAGGTTTGCTGG
**r-Nlrp3-r**	GGTGTAGGGTCTGTTGAGGT
**r-Casp1-f**	GTGGAGAGAAAGAAGGAGTGGT
**r-Casp1-r**	GATGAGTGACTGAATGAAGAGG
**r-GAPDH-f**	GGATAGTGAGAGCAAGAGAGAGG
**r-GAPDH-r**	ATGGTATTGGAGAGAAGGGAGGG

## Conclusion

Maternal treadmill exercise during pregnancy significantly improves cell death in HI offspring. This improvement is likely because of several mechanisms, such as the attenuation of caspase1 and NLRP3 in the HI pups. These findings demonstrated that maternal exercise during pregnancy could be an important tool to improve behavioral and neurological impairments and molecular alterations caused by HI conditions. 

## Limitations and Strengths

The present study had several strengths: 1. Determining the effect of maternal treadmill exercise on the inflammation factor of pups induced by HI injury. 2. Evaluating some neurological outcomes after induced ischemia, including the spontaneous activity, the symmetry of the four limbs, and so on. 3. Histological assessment of the effect of maternal treadmill exercise on HI offspring.

The present study had some limitations: 1) Learning and memory assessment tests were not performed on pups. 2)Neurological functions were not being followed up during the adult period. These limitations limit our ability to generalize conclusions about the effects of maternal treadmill exercise during pregnancy on neonatal HI.

Therefore, it is suggested that more studies examine the effect of maternal treadmill running during pregnancy on the neurobehavioral function of neonatal HI in another period of life.

## Authors’ Contributions

EG designed the study, performed project administration, data analysis, and investigation, and wrote the original draft. HF designed the study, supervised, and performed data analysis. PY designed the study and supervised. HM Performed project administration. MRS carried out the experiments. 

## Ethical Considerations

The study protocol was confirmed by the Faculty of Medicine Ethics Committee for Animal Research of Zahedan University of Medical Sciences (ethical code: IR.ZAUMS.REC.1399.514).

## Funding Sources

Financial support for the study was provided by Elahe Gorgij.

## Data Availability

All data generated in this work will be freely available from the corresponding author upon request.

## Conflicts of Interest

The authors certify no conflicts of interest.
